# Supplementary material for: Networks of necessity: Simulating COVID-19 mitigation strategies for disabled people and their caregivers
Source: PLoS Comput Biol. 2022 May 18;18(5):e1010042. doi: 10.1371/journal.pcbi.1010042 (PMC9232173; doi:10.1371/journal.pcbi.1010042)
Supplement: S1 Text — (PDF) [file pcbi.1010042.s001.pdf]

Networks of Necessity: Simulating COVID-19 Mitigation Strategies for Disabled People and Their Caregivers

Thomas E. Valles[1, 2], Hannah Shoenhard[3], Joseph Zinski[3], Sarah Trick[4], Mason A. Porter[2, 5], and Michael R. Lindstrom[2*]

[1]Department of Mathematics, University of California, San Diego, San Diego, California, United States of America
[2]Department of Mathematics, University of California, Los Angeles, Los Angeles, California, United States of America
[3]Department of Cell and Developmental Biology, University of Pennsylvania, Philadelphia, Pennsylvania, United States of America
[4]Assistant Editor at tvo.org (TVOntario), Toronto, Ontario, Canada
[5]Santa Fe Institute, Santa Fe, New Mexico, United States of America
[*]mikel@math.ucla.edu

May 23, 2022

Supporting Information

A Estimates of Parameter Values

We present the assumptions and derivations that we use to estimate the parameters in our model. (See Section 2 of the main manuscript.) These include parameters that we can obtain directly (possibly with some inference) from the literature and ones that we fit from case data in Ottawa. In our discussion, we use log to denote the natural logarithm.

A.1 Parameters that we Infer from the Literature

A.1.1 Properties of Exponential Distributions

Because we assume that transition times between disease states come from exponential distributions, we state a few useful properties of exponential random variables.

For a random variable X that one samples from an exponential distribution with rate λ (i.e., $X \sim \mathrm{Exp}(\lambda)$), the probability density function is $f(x) = \lambda \mathrm{e}^{-\lambda x}$, the mean is $1/\lambda$, and the median is $\log 2/\lambda$.

Suppose that we have a random variable $Y = \min\{Y_1, Y_2\}$, where Y_1 and Y_2 are random variables that we sample from exponential distributions of rates λ_1 and λ_2, respectively. It then follows that Y is an exponential random variable with rate $\lambda_1 + \lambda_2$ and the probability that $Y_1 < Y_2$ is $\lambda_1/(\lambda_1 + \lambda_2)$.

A.1.2 Parameters

Transition Rate from Exposed to Asymptomatic (ν). The rate of moving from the exposed compartment to a contagious state (and hence to the asymptomatic compartment in our model) has been estimated to be $\nu = 1$ day^{-1} [1].

Recovery Rate from Hospitalization (ζ). The mean duration of hospitalization has been estimated to be $1/\zeta = 12.8$ days [2], so $\zeta \approx 0.0781$ day^{-1}.

Transition Rates from Asymptomatic to Ill (α) and Recovered (η). It has been estimated that 19.45% of cases are entirely asymptomatic [3], so $\frac{\eta}{\eta+\alpha} = 0.1945$. Byrne et al. [4] summarized many relevant studies that give data about different transition rates. From these studies, the mean duration in the asymptomatic state has been estimated to be about $\frac{1}{\alpha+\eta} = 7.25$ days [5] and the median duration has been estimated to be about $\frac{\log 2}{\alpha+\eta} = 9.5$ days [6]. We take the mean of these two values to estimate $\frac{1}{\eta+\alpha} \approx 10.478$ days, which we combine with $\frac{\eta}{\eta+\alpha} = 0.1945$ to obtain $\alpha \approx 0.07688$ day^{-1} and $\eta \approx 0.01856$ day^{-1}.

Transition Rates from Ill to Hospitalized (μ) and Recovered (ρ). It has been estimated that approximately $\frac{\mu}{\mu+\rho} \times 100 = 20\%$ of the symptomatic cases of COVID-19 result in hospitalization [7]. In children with mild cases of COVID-19, the median duration from the onset of symptoms to no longer being infectious is about $\frac{\log 2}{\mu+\rho} = 12$ days [8]. (This study was also referenced in Byrne et al. [4].) In Belgium, the median duration from the onset of symptoms to hospitalization was estimated to be $\frac{\log 2}{\mu+\rho} = 5$ days [9]. We take the mean of the values from these two studies and thereby estimate $\frac{1}{\mu+\rho} \approx 12.2629$ days. With $\frac{\mu}{\mu+\rho} = 0.2$, we obtain $\mu \approx 0.01631$ day^{-1} and $\rho \approx 0.06524$ day^{-1}.

Mask Risk-Reduction Factor (m). Based on three different viruses (SARS CoV-2, SARS-CoV, and MERS-CoV), an unadjusted relative risk of contracting an infection when wearing a face mask versus not wearing one has been reported to be 0.34 (with a 95% confidence window of 0.26 to 0.45) [10]. These results include both healthcare settings and non-healthcare settings. Because the three viruses are from the same family, it was argued in [10] that their relative risks should be comparable. For the data that was reported in this paper, it is not clear if only one or both individuals wore masks in their interactions. We use $m = 0.34$ to represent the risk reduction when both individuals in an interaction wear masks, and we use $\sqrt{m} \approx 0.5831$ if only one individual in an interaction wears a mask. That is, if only one individual in an interaction wears a mask, we quantify the transmission risk as the geometric mean of the best-case transmission reduction if both individuals wear a mask and the worst-case transmission reduction if neither individual wears a mask. By definition, given values $q_1, q_2, \ldots, q_n$, their geometric mean is $(q_1 \times q_2 \times \cdots \times q_n)^{1/n}$. Although our choice seems arbitrary, according to [11], there is a small reduction in the chance of becoming infected in people who wear masks within a household, and it seems plausible that one individual wearing a mask in an interaction between two people confers some reduction in transmission.

Probability of Breaking Weak Contacts if Symptomatic (b). It was very difficult to estimate this parameter. Ultimately, we use the fact that 92% of people in a survey reported practicing physical distancing [12] as a proxy for the fraction of a population who would break their weak contacts if they became symptomatic. That is, $b = 0.92$.

Baseline Transmission Probability β and Caregiving (w_c) and Weak (w_w) Edge Weights. We estimate β and these edge weights based on reported secondary attack rates in various scenarios. The secondary attack rate describes the fraction of a contagious individual's contacts who become infected as a result of interacting with that individual. The secondary attack rate for weak contacts [13] appears to range from about 1% to about 6%, so we estimate it to be 3.5%. Additionally, the secondary attack rate within a household has been estimated to be approximately 20% [13] and is much higher (about 37.8%) between spouses [14].

Caregiving work is extremely intimate and requires extended, close physical contact and potential exposure to bodily fluids. Such a level of intimacy is not typical between housemates, so we use the secondary attack rate between spouses as a proxy for the level of risk in an interaction between a caregiver and a disabled person.

We conduct a set of simulations to estimate the secondary attack rate for each type of contact. The secondary attack rate is the fraction of a contagious individual's contacts that they infect on average. In each trial, we assign a contagiousness duration D_c (which is equal to the asymptomatic time plus any symptomatic time, depending on contact type and on whether or not contacts are broken if an individual becomes ill) and compute the probability that that a contagious individual infects somebody. For weak contacts[1], we use a daily transmission probability of $\sqrt{m} w_w \beta$; for strong contacts, we use a probability of β; for caregiving contacts, we use a probability of $w_c \beta$.

[1]We obtain the value $\sqrt{m}$ by estimating the risk mitigation of masks as the geometric mean of the value (1) when no individual in an interaction wears a mask and the value (m) when both individuals in an interaction wear a mask. We use the geometric mean because of the uncertainty in whether or not people wear masks.

In a single trial, the probability of infection via a strong contact is $1-(1-\beta)^{D_c}$. We then determine the values of w_w, β, and w_c so that, when averaged over many trials, the mean probability of passing on COVID-19 matches the above secondary attack rates. This yields $\beta \approx 0.0112$, $w_w \approx 0.473$, and $w_c \approx 2.268$.

Subpopulation Proportions of the Total Population. By combining the fraction of the population that has a cognitive disability with the fraction that has a physical disability that causes difficulty in dressing, bathing, or getting around inside a home, we estimate that the fraction of our population who are disabled and receive assistance from professional caregivers is $f_{\text{dis}} \approx 0.073$ [15]. Unfortunately, there is a paucity of readily available data, so this is a rough estimate. From the United States Bureau of Labor Statistics, a fraction $f_{\text{care}} \approx 0.021$ of the U.S. population is employed as a home health/professional care aid [16]. We use this number as an estimate of the proportion of the population that provides care. This is likely an underestimate because many people provide care in unpaid settings. From an estimated 55,217,845 essential workers in the United States [17], whose population in July 2019 was estimated to be 328,239,523 [18], the fraction of essential workers is approximately 0.1682. After subtracting the people who are caregivers, we obtain that a fraction $f_{\text{ess}} \approx 0.1472$ of the population are essential workers. That leaves the fraction $f_{\text{gen}} \approx 0.7588$ for the remaining population (i.e., the general population).

Mean Numbers of Contacts. We need distributions of the numbers of family contacts, weak contacts (through work, shopping, seeing friends, and so on), and caregiving contacts. We begin by focusing on the mean values and later consider the distributions themselves. From the 2016 Canadian census [19], households have a mean of 2.4 members, which implies that individuals have a mean of $\bar{F} = 1.4$ strong contacts.

From Gallup data in April 2020 [20], during pandemic lockdowns, the people who were surveyed had a mean of 5.1 contacts per day at work and a mean of 4 contacts per day outside of work and home. Additionally, 27% of working adults completely isolated themselves except to members of their own household. In Europe in 2008, the overall population had a mean of 13.4 daily contacts without a lockdown in place [21]. In April 2020, essential workers saw a mean of 22 contacts per day (this is a much larger number than people who are not essential workers) during the lockdown [22]. By combining these disparate pieces of data, we are able to make some relevant estimates.

Let O_{gd} denote the mean number of occupational contacts of the general and disabled subpopulations on each day without a lockdown, O_{c} denote the mean number of disabled people that a caregiver sees in a day, O^*_{gd} denote the mean number of occupational contacts of the general and disabled subpopulations on each day during a lockdown, w denote the mean number of weak contacts (outside of work) of any individual in a population on each day without a lockdown, w^* denote the mean number of weak contacts (outside of work) of any individual in a population on each day with a lockdown, and O_{e} denote the mean number of occupational contacts of essential workers on each day (both with and without a lockdown). Our parenthetical comment about O_{e} indicates that we are assuming that the number of work contacts is the same for essential workers regardless of whether or not there is a lockdown. We also assume that w does not depend on an individual's subpopulation (disabled person, caregiver, essential worker, or member of the general population). Likewise, we assume that w^* does not depend on an individual's subpopulation.

From the data that we cited two paragraphs ago, we estimate that $w^* = 4$ and that each disabled person sees 2 caregivers per day. Additionally, $O_{\text{c}} = \frac{2f_{\text{dis}}}{f_{\text{care}}} \approx 6.95$ and

$$
\begin{aligned}
22 &\approx \frac{f_{\text{ess}}(O_{\text{e}} + w^* + \bar{F}) + f_{\text{care}}(O_{\text{c}} + w^* + \bar{F})}{f_{\text{ess}} + f_{\text{care}}} \\
5.1 &\approx (f_{\text{care}} + f_{\text{dis}})O^*_{\text{gd}} + f_{\text{ess}}O_{\text{e}} + f_{\text{care}}O_{\text{c}} \\
13.4 &\approx f_{\text{gen}}(O_{\text{gd}} + w + \bar{F}) + f_{\text{dis}}(O_{\text{gd}} + w + \bar{F}) + f_{\text{ess}}(O_{\text{e}} + w + \bar{F}) + f_{\text{care}}(O_{\text{c}} + w + \bar{F})\,.
\end{aligned}
$$

To close the system of equations and obtain our estimates, we require one further assumption. If 27% of workers isolate at home, then the mean number of contacts at work is

$$O^*_{\text{gd}} \approx [0.27 \times 0] + [0.73 \times (0.73\,O_{\text{gd}})] \approx 0.5329\,O_{\text{gd}}\,.$$

We obtain $w \approx 5.14$, $w^* \approx 4$, $O_{\text{e}} \approx 16.23$, $O_{\text{c}} \approx 6.95$, $O_{\text{gd}} \approx 5.20$, and $O^*_{\text{gd}} \approx 3.08$. When we use approximate truncated power-law distributions to model the possibility that some people have many contacts and others have few contacts, we want to satisfy the following criteria:

- the general population has a mean of $w + O_{\text{gd}} \approx 10.34$ weak contacts per day when not physically distancing and a mean of $w^* + O^*_{\text{gd}} \approx 7.08$ weak contacts per day when physically distancing;

- the disabled subpopulation has the same mean value of weak contacts as the general population whether or not people are physically distancing;
- the caregiver subpopulation has a mean of $w \approx 5.14$ weak contacts per day when not physically distancing and a mean of $w \approx 4$ weak contacts per day when physically distancing; and
- the essential-worker subpopulation has a mean of $O_{\mathrm{e}} + w \approx 21.37$ weak contacts per day when not physically distancing and a mean of $O_{\mathrm{e}} + w^* \approx 20.23$ contacts per day when physically distancing.

Although the caregiver subpopulation may seem to have very few weak contacts, we note that most of their daily contacts come from O_{c}, which we estimate separately from the ordinary weak contacts.

Although the number of weak contacts for essential workers does decrease slightly during a lockdown, we use $O_{\mathrm{e}} + w$ whether or not a lockdown is in place as an approximation because the difference in the numbers of weak contacts is very small (21.37 versus 20.23). In practice, it was difficult for us to reduce the mean number of contacts by such a small amount in this situation. Picking the minimum of two random variables from similar distributions tends to result in a value that is much smaller than the original one and thereby results in the essential workers having far too few contacts.

Distribution of Strong Contacts: We use data from the 2016 Canadian census [19] to describe the distribution of household sizes. According to these data, 105,750 households consist of 1 person, 124,280 households consist of 2 people, 58,010 households consist of 3 people, 55,215 households consist of 4 people, and 30,500 households consist of 5 or more people (which we treat as exactly 5 people). From these data, we construct an empirical distribution that we use for the entire population. It is $\mathcal{D}_s = \mathcal{E}(0.283, 0.332, 0.155, 0.148, 0.0816)$.

Caregivers: To each disabled person, we assign one strong caregiver and one weak caregiver with whom they interact each day (although they do not interact with the latter when either they or the caregiver is symptomatic). We choose the weak caregivers from a pool of caregivers. We use 10 as the baseline caregiver-pool size, but we also consider other sizes (4 and 25, as we discussed in Section 3 of the main manuscript).

A.2 Fits from Data

We need to estimate three other parameters in our model. Even with our many estimates from the literature that we discussed in Section A.1, we still need to estimate the following quantities: (1) the maximum number C^* of weak contacts of an individual, (2) the number A_0 of people who are asymptomatic on day 0, and (3) the probability τ that an individual who is symptomatically ill but not hospitalized is counted in the cumulative number of cases.

We model the number of weak contacts using an approximate truncated power-law distribution. That is, the daily number of weak contacts of an individual is distributed according to $\mathcal{P}(0, C^*; O_q)$, where O_q denotes the mean number of weak contacts of subpopulation q.

Along with the simulation procedure that we will describe in Section B, we use a fitting procedure (along with case data from Ottawa [23]) to estimate τ and C^* with a grid search. We use the first 90 days as fitting data and assume that the associated contact distributions and mask-wearing policies are instantly adopted on day 44 (i.e., the start of the lockdown in Ottawa). We tried fitting over shorter time windows, but these yielded poorer fits. The likely reason for the poor fits for these shorter time windows is that the parameter C^* is smaller when fit over shorter time intervals (because the disease has spread less at that stage). The longer time window allows us to fit C^*, which may be a key driver in the disease dynamics, to a larger value and thereby allows extensive spreading of the disease.

We assume that there are A_0 people on day 0 in the asymptomatic compartment and that all other individuals are in the susceptible compartment. On day 1, with the first recorded case, there is 1 recorded case in expectation. Therefore,

$$1 = \underbrace{\frac{\alpha}{\alpha + \eta}}_{\text{Pr(transition from asymptomatic to ill)}} \times \underbrace{\mathrm{e}^{-(\alpha+\eta)\times 1\,\mathrm{day}}}_{\text{Pr(leave the A compartment within 1 day)}} \times \underbrace{\tau}_{\text{test ill individual}} \times A_0 \,. \tag{1}$$

The first factor is the probability that the transition from the A compartment to the I compartment occurs before the transition from A to the R compartment. The second factor is the probability that there is a transition out of the A compartment in a 1-day time period. The third factor is the probability that an individual in the I compartment tests positive for COVID-19. The fourth factor (A_0) is the total number of asymptomatic people on day 0. Our choice to

make the expected number of documented cases equal to 1 on day 1 allows us to have two parameters (rather than three) when fitting. Using more parameters can result in overfitting.

We seek to minimize the ℓ_2-error in new daily cases (i.e., the change in the daily cumulative case count). Because our stochastic model is complicated, with variation across trials, we use a grid search (instead of a gradient-based method) to estimate parameters. In Table A, we summarize our results. From this procedure, our "optimal" parameter values are $\tau = 0.04$ and $C^* = 60$.

B Simulations of our Stochastic Model of COVID-19 Spread

B.1 Simulation Procedure

We summarize our simulation procedure in Algorithm 1, which uses the other algorithms that we present in this subsection. The code is available at our Bitbucket repository.

We initially construct a network by matching ends of edges (i.e., "stubs") in a generalization of a configuration-model network. We assign a number of stubs to each individual in each subpopulation to encode their number of weak contacts (see Algorithm 2). We determine this number from an associated probability distribution. We then do a so-called "random matching" (see Algorithm 4), in which we match stubs uniformly at random. Any pair of individuals whose stubs are matched in this way are contacts of each other. If we choose two individuals who are already contacts or an individual is paired with themself, we discard that pairing. For strong contacts, we assign individuals to units (see Algorithm 3) and make members of these units strong contacts with each other unless they are already contacts (see Algorithm 5). Consequently, the number of contacts per individual does not perfectly match the desired distributions. However, for a network with many nodes, these errors are negligible in practice. See [24] for a detailed exposition of different types of configuration models (although we employ a generalization of a configuration model), including different strategies for how to deal with self-edges and multi-edges. We assign weak and strong caregivers to disabled people in a manner (see Algorithm 6) that is analogous to how we assign strong contacts.

After constructing a contact network, we place some number of individuals, who we choose uniformly at random from the nodes in the network, into the A and/or I compartments. This number of individuals, the subpopulations of these individuals, and the choice of these compartments (all of these individuals in A, all of these individuals in I, or some of these individuals in A and some of them in I) depend on user input. For example, in the four simulations that we used to generate Fig 10 in the main manuscript, all initially infected individuals are in the A compartment and are in a single subpopulation (caregivers, disabled people, essential workers, or the general population). In all other simulations that we discuss in the present paper, the initially infected individuals are all in the A compartment.

After initializing the contact structures and the compartments of the nodes, we execute the commands in the following paragraphs for a user-specified number of iterations.

We check if we need to update contact structures and/or mask-wearing strategies because of a lockdown (see Algorithm 9) or a reopening (see Algorithm 10). For a lockdown, we update the mask-wearing strategies and assign each individual a number of weak contacts from the new weak-contact distribution that is associated with their subpopulation. If the new number of weak contacts is smaller than the current number of weak contacts, we remove excess contacts uniformly at random. For a reopening, we again update the mask-wearing strategies and assign each individual a number of weak contacts from their subpopulation's new weak-contact distribution. If the new number of weak contacts is larger than the current number of weak contacts, we assign the individual a number of stubs that is equal to the difference between the new number of contacts and the existing number of contacts and apply Algorithm 4 to connect the stubs.

On each day, we assign a weak caregiver to each disabled person uniformly at random from their pool of weak caregivers, as long as neither is breaking their contacts. We then use Algorithm 8 to determine if each individual in the network remains in their current compartment or moves to a new one. If an individual is in the S compartment, we calculate the probability of infection using Algorithm 7. In this algorithm, we loop through each of this individual's contagious contacts (i.e., those in the A, I, or H compartments) and use Eq. (1) of the main manuscript to calculate the probability that the individual becomes infected. For the E and H compartments, for which there is only one possible transition to a new compartment, we draw a transition time from an exponential distribution $\text{Exp}(\chi)$ (where χ is the associated rate constant) to determine if there is a transition between compartments. If the time is less than 1 day, then the individual changes compartments; otherwise, the individual stays in their current compartment. For the A and I compartments, from which an individual can move into one of two possible new compartments, we draw transition times from $\text{Exp}(\chi_1)$ and $\text{Exp}(\chi_2)$, where χ_1 and χ_2 are the associated rate constants. If both times are less than 1 day, the

τ	C^*	Error
0.02	50	2.31×10^4
0.03	50	1.82×10^4
0.03	60	2.38×10^4
0.04	50	2.13×10^4
**0.04**	**60**	$\mathbf{1.74 \times 10^4}$
0.04	70	3.05×10^4
0.05	50	2.48×10^4
0.05	60	1.83×10^4
0.05	70	2.26×10^4
0.06	50	2.80×10^4
0.06	60	2.01×10^4
0.06	70	1.76×10^4
0.06	80	3.48×10^4
0.07	50	3.16×10^4
0.07	60	2.26×10^4
0.07	70	1.80×10^4
0.07	80	2.78×10^4
0.08	50	3.32×10^4
0.08	60	2.38×10^4
0.08	70	1.86×10^4
0.08	80	2.51×10^4
0.09	50	3.55×10^4
0.09	60	2.69×10^4
0.09	70	1.93×10^4
0.09	80	2.25×10^4
0.10	50	3.61×10^4
0.10	60	2.82×10^4
0.10	70	2.07×10^4
0.10	80	1.96×10^4
0.10	90	4.40×10^4
0.11	50	3.80×10^4
0.11	60	2.88×10^4
0.11	70	2.16×10^4
0.11	80	1.99×10^4
0.11	90	3.54×10^4

Table A. The ℓ_2-error in new daily documented cases for various values of τ and C^*. To determine A_0, we use Eq. (1) of this supplement with a specified value of τ and values of α and μ from the literature. For each set of parameters, we conduct 96 trials and we compute the error by taking the mean of all trials in which there are at least 250 documented cases through day 90. We only report parameter values for which the errors are smaller than 5×10^4. We test all parameter values on the lattice with coordinates $(\tau, C^*) \in \{0.01, 0.02, 0.03, 0.04, 0.05, 0.06, 0.07, 0.08, 0.09.0.10, 0.11\} \times \{50, 60, 70, 80, 90, 100, 110, 120, 130, 140, 150\}$. We show our best results in bold. That is, our "optimal" parameter values (see the fifth row) are $\tau = 0.04$ and $C^* = 60$.

individual moves to the compartment that has the smaller time. If only one of the times is less than 1 day, the individual moves to that compartment. If neither time is less than 1 day, the individual remains in their current compartment. When an individual enters the I compartment, they may break their weak contacts. With probability b, they break all of their weak contacts; otherwise, they keep all of their weak contacts. Individuals in the I compartment become documented cases with probability τ. In our pseudocode, we refer to the breaking of contacts as "deactivating" edges and refer to the re-establishment of contacts as "reactivating" edges. If an individual moves to the H compartment, we deactivate all of their edges with weak and strong contacts. If an individual moves to the R compartment, we reactivate any edges that may have been deactivated because of their movement through the I and H compartments (except edges that are not active because (1) the other individual in the interaction is in the I compartment and broke their weak contact or (2) the other individual is in the H compartment).

B.2 Implementation of Approximate Truncated Power-Law Distributions

B.2.1 Sampling from the Distribution

Given a lower bound a_-, an upper bound a_+, and an exponent p, we wish to approximate a power-law distribution for a discrete random variable N over the interval $[a_-, a_+]$, where $\Pr(N = n) = O(n^{-p})$ as $a_+, n \to \infty$. Our procedure amounts to (1) shifting the range to avoid the case $a_- = 0$, (2) sampling from a continuous power-law probability density, (3) truncating the result to an integer, and (4) shifting the range back if we shifted the original range away from $a_- = 0$. In our model, we use $a_- = 0$ and $a_+ = C^*$, but we present the approach for a general finite sequence of nonnegative integers.

If $a_- = 0$, we first shift to a distribution on $[A, B]$, where $A = \max\{a_-, 1\}$ and $B = a_+ + (A - a_-)$. We define the normalization constant

$$C = \int_A^{B+1} x^{-p}\,\mathrm{d}x = \begin{cases} \frac{1}{1-p}\left((B+1)^{1-p} - A^{1-p}\right), & p \neq 1 \\ \log\left(\frac{B+1}{A}\right), & p = 1\,. \end{cases} \tag{2}$$

(Note that one should not conflate C with C^*.) To choose N, we select $u \in [0, 1)$ from a uniform distribution and select x^* such that

$$C^{-1} \int_A^{x^*} x^{-p}\,\mathrm{d}x = u\,. \tag{3}$$

We then calculate

$$n^* = \lfloor x^* \rfloor\,, \tag{4}$$

where $\lfloor z \rfloor$ is the floor of z (i.e., the largest integer that is less than or equal to z). That is,

$$x^* = \begin{cases} \left((1-p)uC + A^{1-p}\right)^{1/(1-p)}, & p \neq 1 \\ A\exp(uC), & p = 1\,. \end{cases} \tag{5}$$

Finally, we shift back to set

$$N = n^* - (A - a_-)\,. \tag{6}$$

Note that

$$\begin{aligned} \Pr(N = n) \propto \int_{n+(A-a_-)}^{n+1+(A-a_-)} x^{-p}\,\mathrm{d}x &= \begin{cases} \log\left(\frac{n+1+(A-a_-)}{n+(A-a_-)}\right), & p = 1 \\ \left|(n+1+(A-a_-))^{1-p} - (n+(A-a_-))^{1-p}\right|, & p \neq 1 \end{cases} \\ &= \begin{cases} \log\left(1 + \frac{1}{n+(A-a_-)}\right), & p = 1 \\ \left|(n+(A-a_-))^{1-p}\left(1 + \frac{1}{N+(A-a_-)}\right)^{1-p} - 1\right|, & p \neq 1 \end{cases} \\ &= O(1/n^p) \quad \text{as} \quad n \to \infty\,, \end{aligned}$$

thereby ensuring that asymptotically we have a power law as $n \to \infty$.

Algorithm 1 A Simulation of the Spread of COVID-19 on a Contact Network

Input: A set of values for each parameter that we list in Table 1 of the main manuscript
Output: Daily counts of the individuals in each compartment; number of documented cases

Initialize *Population* of size P_{Ottawa} with fractions f_{dis} who are disabled, f_{care} who are caregivers, f_{ess} who are essential workers, and f_{gen} who are members of the general population. At initialization, we determine whether or not each individual will break all of their weak contacts if they become ill (they break weak contacts with probability b) and determine whether or not they will have a positive test result if they become ill (a positive test occurs with probability τ).
Assign a unique integer ID to each individual in *Population*.
Obtain *WeakStubs* from Algorithm 2 with input *Population*.
Obtain *PossibleHouseholdUnits* from Algorithm 3 with input *Population*.
Assign weak contacts using Algorithm 4 with inputs *Population*, *WeakStubs*.
Assign strong contacts using Algorithm 5 with inputs *Population*, *PossibleHouseholdUnits*.
Match disabled people and caregivers using Algorithm 6 with input *DisabledPopulation*, where *DisabledPopulation* refers to all individuals in *Population* who are in the disabled subpopulation.
Initialize some number of people to be asymptomatic or ill based on program inputs. (In all of our simulations in the present paper, we initialize these individuals to be asymptomatic, but one can instead use our code to initialize individuals as ill; one can also initialize some individuals to be asymptomatic and some individuals to be ill.)
$day = 0$, $has\_opened =$ **false**, $has\_closed =$ **false**
while $day < end\_day$ **do**
    Compute the number of individuals from each subpopulation in each compartment; also compute the number of documented cases in each subpopulation.
    for each *disabled_individual* in *DisabledPopulation* **do**
        Select a weak caregiver uniformly at random from their set of weak caregivers.
    end for
    for each *individual* in *Population* **do**
        Calculate the infection probability using Algorithm 7 with input *individual*.
    end for
    for each *individual* in *Population* **do**
        Advance state by 1 day using Algorithm 8 with input *individual*.
    end for
    $day \leftarrow day + 1$
    if $time < close\_time$ **then**
        Do nothing.
    else if $time < open\_time$ **then**
        if not *has_closed* **then**
            Close down (i.e., start a lockdown) using Algorithm 9.
            $has\_closed \leftarrow$ **true**
        end if
    else
        if not *has_opened* **then**
            Reopen (i.e., end a lockdown) using Algorithm 10.
            $has\_opened \leftarrow$ **true**
        end if
    end if
end while

Algorithm 2 Weak Stubs

Input: A container of nodes (which we denote by *Population*)

Output: A container of IDs (which we denote by *WeakStubs*) in which the ID of each node in *Population* occurs with a multiplicity that is equal to the number of stubs of that node.

```
for each individual in Population do
    Let target equal the number of weak stubs that individual can potentially have; we draw this number from D_{group,period}, where "group" is their subpopulation and "period" is the current state of the pandemic (pre-lockdown, lockdown, or post-lockdown).
    Let current equal the number of current weak stubs of individual.
    if current < target then
        needed = current − target
    else
        needed = 0
    end if
    For needed number of times, append the ID of individual to a container WeakStubs.
    return WeakStubs
end for
```

Algorithm 3 Household Units

Input: A container of nodes (which we denote by *Population*)

Output: A container of containers of IDs (which we denote by *PossibleHouseholdUnits*)

```
Let AllIDs be a container that stores the unique ID for each individual in Population.
while AllIDs not empty do
    Choose an ID, which we denote by ID_1, uniformly at random from AllIDs and determine the number of household contacts (house) of the individual with that ID by sampling from D_strong.
    Select house number of IDs uniformly at random from AllIDs.
    Append ID_1 and the above IDs to a container, which we denote by unit.
    Remove all of the IDs in unit from AllIDs.
end while
return PossibleHouseholdUnits (which is a container that holds each unit)
```

Algorithm 4 Assigning Weak Contacts

Input: A container of nodes (which we denote by *Population*) and a container of IDs (which we denote by *WeakStubs*)

Result: All nodes in *Population* are assigned weak contacts

```
while |WeakStubs| ≥ 2 do
    Choose IDs ID_1 and ID_2 uniformly at random from WeakStubs.
    if ID_1 ≠ ID_2 and the individuals with IDs ID_1 and ID_2 are not already contacts (weak, strong, or caregiving) then
        Make the individuals with IDs ID_1 and ID_2 into weak contacts of each other.
    end if
    Remove ID_1 and ID_2 from WeakStubs.
end while
```

Algorithm 5 Assigning Strong Contacts

Input: A container of nodes (which we denote by *Population*) and a container of containers of IDs (which we denote by *PossibleHouseholdUnits*)
Result: All nodes in *Population* are assigned strong contacts

```
for each unit in PossibleHouseholdUnits do
    for each ID in unit do
        Make the individual with ID a strong contact of each other member of unit, unless the individuals are already
        contacts (weak, strong, or caregiving).
    end for
end for
```

Algorithm 6 Matching Disabled People and Caregivers

Input: A container of nodes (which we denote by *DisabledPopultion*) that are in the same subpopulation
Result: All nodes in *DisabledPopulation* are assigned one strong caregiver and a pool of weak caregivers

```
for each disabled_individual in DisabledPopulation do
    Determine care_weak_num from D_pool, which is the number of weak caregivers in their pool.
    Select care_weak_num number of caregivers uniformly at random from the set of caregivers and store them in
    CaregiversChosen.
    for each caregiver in CaregiversChosen do
        if disabled_individual and caregiver are not already contacts (weak, strong, or caregiving) then
            Make their relationship a weak caregiver–disabled relationship.
        end if
    end for
end for
for each disabled_individual in DisabledPopulation do
    Choose 1 caregiver uniformly at random from the set of caregivers.
    if disabled_individual and the caregiver are not already contacts (weak, strong, or caregiving) then
        Make their relationship a strong caregiver–disabled relationship.
    end if
end for
```

Algorithm 7 Infection Probability

Input: A node (which we denote by *individual*)

Output: An infection probability (which we denote by *infect_prob*)

```
not_get = 1
if individual is Susceptible then
  for each weak_contact in individual's weak contacts do
    if the edge to weak_contact is active and weak_contact is contagious then
      if both wear a mask then
        not_get ← not_get × (1 − \beta m w_w)
      else
        not_get ← not_get × (1 − \beta w_w)
      end if
    end if
  end for
  for each strong_contact in individual's strong contacts do
    if the edge to strong_contact is active and strong_contact is contagious then
      not_get ← not_get × (1 − \beta w_s)
    end if
  end for
  if individual is disabled then
    for each caregiver in their set of weak caregivers for the day do
      if the edge to caregiver is active and caregiver is contagious then
        if both wear a mask then
          not_get ← not_get × (1 − \beta m w_c)
        else if one wears a mask then
          not_get ← not_get × (1 − \beta \sqrt{m} w_c)
        else
          not_get ← not_get × (1 − \beta w_c)
        end if
      end if
    end for
    if the edge to individual's strong caregiver is active and the strong caregiver is contagious then
      if both wear a mask then
        not_get ← not_get × (1 − \beta m w_c)
      else if one wears mask then
        not_get ← not_get × (1 − \beta \sqrt{m} w_c)
      else
        not_get ← not_get × (1 − \beta w_c)
      end if
    end if
  else if individual is a caregiver then
    for each disabled_individual in their set of disabled contacts for the day do
      if the edge to disabled_individual is active and disabled_individual is contagious then
        if both wear a mask then
          not_get ← not_get × (1 − \beta m w_c)
        else if one wears mask then
          not_get ← not_get × (1 − \beta \sqrt{m} w_c)
        else
          not_get ← not_get × (1 − \beta w_c)
        end if
      end if
    end for
  end if
end if
infect_prob = 1 − not_get
return infect_prob
```

Algorithm 8 Advancing One Day

Input: A node (which we denote by *individual*)
Result: *individual* remains in their current compartment or moves into a new one

if *individual* is in the S compartment **then**
In the time interval $\Delta T = 1$ day, move *individual* into the E compartment with probability *infect_prob.*
else if *individual* is in the E compartment **then**
Sample $T_{\text{asymptomatic}}$ from the distribution $\text{Exp}(\nu)$.
if $T_{\text{asymptomatic}} < 1$ day **then**
Move *individual* to the A compartment.
end if
else if *individual* is in the A compartment **then**
Sample T_{ill} from $\text{Exp}(\alpha)$.
Sample T_{removed} from $\text{Exp}(\eta)$.
if $T_{\text{ill}} < T_{\text{removed}}$ **then**
if $T_{\text{ill}} < 1$ day **then**
Move *individual* to the I compartment.
Deactivate all of *individual*'s edges to weak contacts if *individual* is someone who breaks their weak contacts.
end if
else
if $T_{\text{removed}} < 1$ day **then**
Move *individual* to the R compartment.
Reactivate all of *individual*'s edges to their weak contacts (provided, for each weak contact, either that the weak contact has no symptoms or that they are in the I compartment but do not break weak contacts when ill).
end if
end if
else if *individual* is in the I compartment **then**
Sample T_{hospital} from $\text{Exp}(\mu)$.
Sample T_{removed} from $\text{Exp}(\rho)$.
if $T_{\text{hospital}} < T_{\text{removed}}$ **then**
if $T_{\text{hospital}} < 1$ day **then**
Move *individual* to the H compartment.
Deactivate all of *individual*'s edges to their weak and strong contacts.
end if
else
if $T_{\text{removed}} < 1$ day **then**
Move *individual* to the R compartment.
Reactivate all of *individual*'s edges to their weak contacts (provided, for each weak contact, either that the weak contact has no symptoms or that they are in the I compartment but do not break weak contacts when ill).
end if
end if
else if *individual* is in the H compartment **then**
Sample T_{removed} from $\text{Exp}(\zeta)$.
if $T_{\text{removed}} < 1$ day **then**
Move *individual* to the R compartment.
Reactivate all of *individual*'s edges to their weak contacts (provided, for each weak contact, either that the weak contact has no symptoms or that they are in the I compartment but do not break weak contacts when ill).
end if
end if

Algorithm 9 Closing Down (i.e., Starting a Lockdown)

Input: A container of nodes (which we denote by *Population*)

Result: Lockdown mask-wearing strategies and contact-limiting strategies are implemented for each node in *Population*

Update mask-wearing statuses.
for each *individual* in *Population* **do**
    Determine *individual*'s new number of weak contacts by sampling *new_target_value* from $\mathcal{D}_{\text{group,post}}$, where *group* is the subpopulation of the individual.
end for
for each *individual* in *Population* **do**
    $\mathit{clear} = \max\{0, \mathit{current\_weak\_contacts} - \mathit{new\_target\_value}\}$
    $i = 0$
    while $i < \mathit{clear}$ **do**
        Select a weak contact ϖ uniformly at random.
        if neither ϖ nor *individual* is an essential worker **then**
            Remove the edge between the nodes.
        end if
        $i \leftarrow i + 1$
    end while
end for

Algorithm 10 Reopening (i.e., Ending a Lockdown)

Input: A container of nodes (which we denote by *Population*)

Output: Reopening mask-wearing strategies and contact-limiting strategies are implemented for each node in *Population*

Update mask-wearing statuses.
Obtain a container *new_weak_stubs* by applying Algorithm 2 with input *Population*.
Apply Algorithm 4 with inputs *Population* and *new_weak_stubs*.

B.2.2 Estimating the Mean

When $B-A$ is large, it can be computationally expensive to compute the precise mean of the random variable $N = n^* - (A - a_-)$ from Eq. (6) of this supplement. Additionally, when $B-A$ is large, rounding errors and overflow errors can lead to inaccurate estimations of the true mean. Therefore, we estimate the mean analytically. Given a_-, a_+, and p, we seek to estimate the mean $E_p := \mathbb{E}(N)$ over the interval $[a_-, a_+]$.

We have that

$$\begin{aligned} E_p &= C^{-1} \sum_{n=A}^{B} n \int_n^{n+1} x^{-p}\, \mathrm{d}x \\ &= \begin{cases} C^{-1} \sum_{n=A}^{b} n \log((n+1)/n)\,, & p = 1 \\ ((1-p)C)^{-1} \sum_{n=A}^{b} n((n+1)^{1-p} - n^{1-p})\,, & p \neq 1 \end{cases} \\ &= \begin{cases} C^{-1} \sum_{n=A}^{b} n \log((n+1)/n)\,, & p = 1 \\ ((1-p)C)^{-1} \left((B+1)^{2-p} - A^{2-p} - \sum_{n=A+1}^{B+1} n^{1-p} \right), & p \neq 1\,. \end{cases} \end{aligned}$$

To obtain the third equality, we rewrote $\sum_{n=A}^{B} n(n+1)^{1-p}$ as $\sum_{n=A+1}^{B+1}(n-1)n^{1-p}$, whose n^{2-p} terms cancel with $\sum_{n=A}^{B} n^{2-p}$ except at $n = A$ and $n = B+1$.

For our approximation, we consider multiple cases.

$p = 1$: Because $\{n \log(\frac{n+1}{n})\}_{n=A}^{B}$ is an increasing sequence of terms, we obtain

$$\underline{S}_1 := A \log\left(\frac{A+1}{A}\right) + \int_A^B x \log\left(\frac{x+1}{x}\right) \mathrm{d}x \leq \sum_{n=A}^{B} n \log\left(\frac{n+1}{n}\right) \leq \int_A^{B+1} x \log\left(\frac{x+1}{x}\right) \mathrm{d}x =: \overline{S}_1\,.$$

Using $\int x \log(\frac{x+1}{x})\, \mathrm{d}x = \frac{1}{2}(x^2 \log((x+1)/x) + x - \log(x+1)) + \text{const}$, we compute the integrals exactly and obtain the estimate $E_1 = C^{-1}(\underline{S}_1 + \overline{S}_1)/2$.

$p = 2$: We need to estimate $\sum_{n=A+1}^{B+1} n^{-1}$. The sequence $1/n$ is decreasing, so

$$\underline{S}_2 := \int_{A+1}^{B+2} \frac{1}{x}\, \mathrm{d}x = \log\left(\frac{B+2}{A+1}\right) \leq \sum_{n=A+1}^{B+1} n^{-1} \leq \frac{1}{A+1} + \log\left(\frac{B+1}{A+1}\right) = \frac{1}{A+1} + \int_{A+1}^{B+1} \frac{1}{x}\, \mathrm{d}x =: \overline{S}_2\,.$$

We obtain the estimate $E_2 = C^{-1}\left(\frac{1}{2}(\underline{S}_2 + \overline{S}_2)\right)$, where $A^{2-p} = (B+1)^{2-p} = 1$ allows us to cancel terms.

$p \notin \{1, 2\}\,,\, p > 1$: We need to estimate $\sum_{n=A+1}^{B+1} n^{1-p}$, where the terms are decreasing. Therefore,

$$\begin{aligned} \underline{S}_{p>} &:= \int_{A+1}^{B+2} x^{1-p}\, \mathrm{d}x = \frac{(B+2)^{2-p} - (A+1)^{2-p}}{2-p} \leq \sum_{n=A+1}^{B+1} n^{1-p} \\ &\leq (A+1)^{1-p} + \frac{(B+1)^{2-p} - (A+1)^{2-p}}{2-p} = (A+1)^{1-p} + \int_{A+1}^{B+1} x^{1-p}\, \mathrm{d}x =: \overline{S}_{p>}\,. \end{aligned}$$

We obtain the estimate $E_{p>} = ((1-p)C)^{-1}\left((B+1)^{2-p} - A^{2-p} - \frac{1}{2}(\underline{S}_{p>} + \overline{S}_{p>})\right)$.

$p \notin \{1, 2\}\,,\, p < 1$: We need to estimate $\sum_{n=A+1}^{B+1} n^{1-p}$, where the terms are increasing. Therefore,

$$\begin{aligned} \underline{S}_{p<} &:= (A+1)^{1-p} + \int_{A+1}^{B+1} x^{1-p}\, \mathrm{d}x = (A+1)^{1-p} + \frac{(B+1)^{2-p} - (A+1)^{2-p}}{2-p} \leq \sum_{n=A+1}^{B+1} n^{1-p} \\ &\leq \frac{(B+2)^{2-p} - (A+1)^{2-p}}{2-p} = \int_{A+1}^{B+2} x^{1-p}\, \mathrm{d}x =: \overline{S}_{p<}\,. \end{aligned}$$

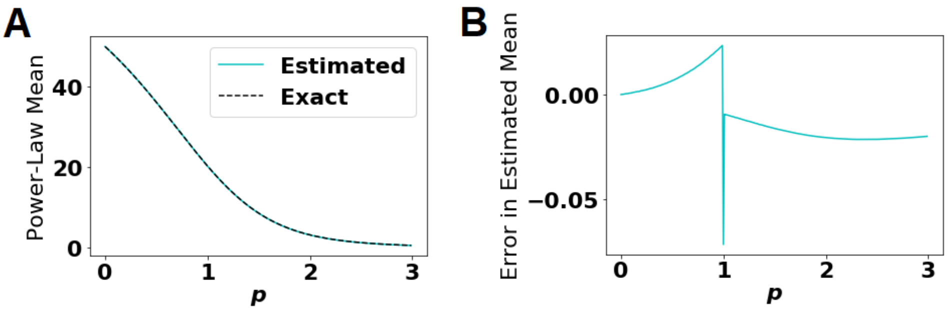

S1 Fig. (A) The estimated and exact mean values of the approximate truncated power-law distribution for various values of the exponent p. The curves are indistinguishable. (B) The error in computing the mean for our approximations. In both panels of this figure, we use $a_{-} = 0$ and $a_{+} = 100$.

We obtain the estimate $E_{p<} = ((1-p)C)^{-1} \left((B+1)^{2-p} - A^{2-p} - \frac{1}{2}(\underline{S}_{p<} + \overline{S}_{p<})\right)$.

Our approximation is very accurate. For $a_{-} = 0$ and $a_{+} = 100$, we plot the approximations and the numerically exact values in S1 Fig.

C Additional Computational Experiments

C.1 Examining a Distribution with a Deterministic Number of Weak Contacts

The confidence windows for the cumulative documented case counts are large. To determine the cause of these large variances, we run trials (see S2 Fig) in which each subpopulation has a deterministic number of weak contacts that is equal to the associated mean value in Section 2.2 of the main manuscript. When the weak-contact distribution is deterministic, we obtain much smaller variances in the numbers of documented cases than when weak contacts are distributed according to an approximate truncated power-law. Additionally, using a deterministic distribution results in many fewer cases of the disease, which hardly spreads.

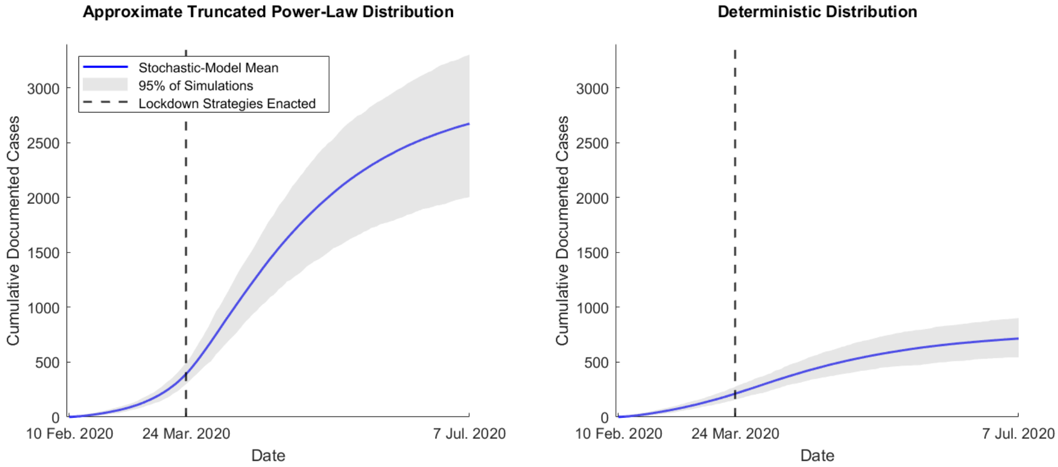

S2 Fig. Comparison of a mean of 100 simulations when the weak contacts follow (left) an approximate truncated power-law distribution and (right) a deterministic distribution. In both plots, the mean is in blue and the gray window indicates the middle 95% of the 100 simulations. On day 44 (i.e., 24 March 2020), all groups limit contacts, all individuals in caregiver–disabled interactions wear masks, and all individuals wear masks in interactions between essential workers and their weak contacts.

C.2 Different Values of the Caregiver–Disabled Edge Weight w_c

The risk of COVID-19 infections in a caregiver–disabled interaction is larger than in an ordinary household interaction. In S3 Fig, we compare our results for two different values of the caregiver–disabled edge weight w_c. The choice $w_c = 1$ results in essential workers, who have many weak contacts, being the most potent disease spreaders of the four examined subpopulations (except for spreading from caregivers to other caregivers). However, even the choice $w_c = 1.5$ (which is smaller than the value $w_c = 2.27$ that we used in most of our computations) results in caregivers being the most potent spreaders of the disease to the disabled subpopulation.

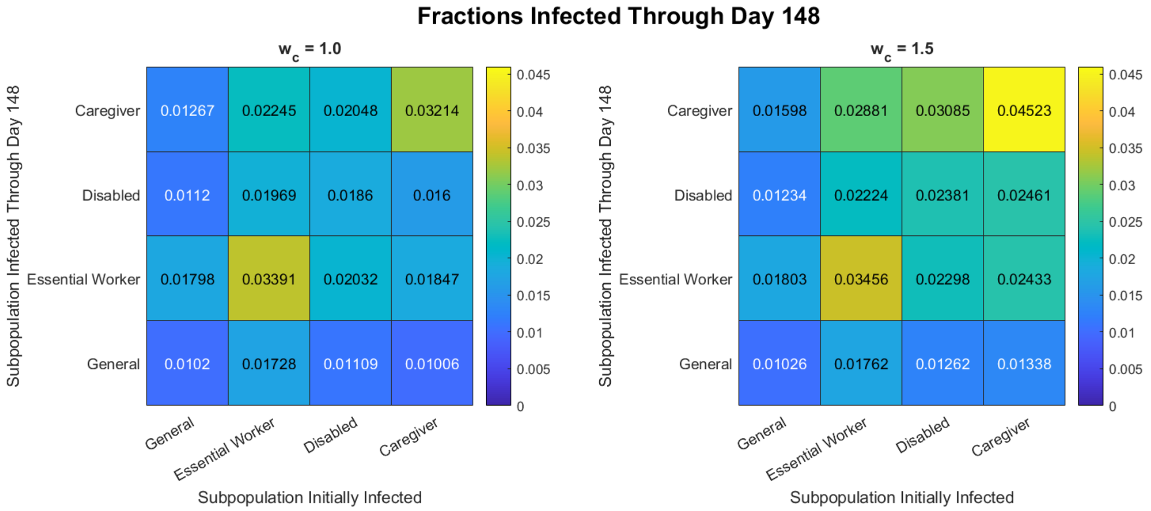

S3 Fig. The fraction of each subpopulation that is infected through day 148 when all of the initially infected individuals are in a single subpopulation for (left) $w_c = 1$ and (right) $w_c = 1.5$. On day 44, all groups limit contacts and disabled people, caregivers, and essential workers wear masks.

References

1. Anderson RM, Heesterbeek H, Klinkenberg D, Hollingsworth TD. How Will Country-Based Mitigation Measures Influence the Course of the COVID-19 Epidemic? The Lancet. 2020;395(10228):931–4. Available from: `https://doi.org/10.1016/S0140-6736(20)30567-5`.

2. Guan Wj, Ni Zy, Hu Y, et al. Clinical Characteristics of Coronavirus Disease 2019 in China. New England Journal of Medicine. 2020;382(18):1708–20. Available from: `https://www.nejm.org/doi/full/10.1056/NEJMoa2002032`.

3. Buitrago-Garcia D, Egli-Gany D, Counotte MJ, et al. Occurrence and Transmission Potential of Asymptomatic and Presymptomatic SARS-CoV-2 Infections: A Living Systematic Review and Meta-Analysis. PLoS Medicine. 2020;17(9):e1003346. Available from: `https://doi.org/10.1371/journal.pmed.1003346`.

4. Byrne AW, McEvoy D, Collins AB, et al. Inferred Duration of Infectious Period of SARS-CoV-2: Rapid Scoping Review and Analysis of Available Evidence for Asymptomatic and Symptomatic COVID-19 Cases. BMJ Open. 2020;10(8):e039856. Available from: `10.1136/bmjopen-2020-039856`.

5. Ma S, Zhang J, Zeng M, et al. Epidemiological Parameters of Coronavirus Disease 2019: A Pooled Analysis of Publicly Reported Individual Data of 1155 Cases From Seven Countries. MedRxiv. 2020. Available from: `doi:https://doi.org/10.1101/2020.03.21.20040329`.

6. Hu Z, Song C, Xu C, et al. Clinical Characteristics of 24 Asymptomatic Infections With COVID-19 Screened Among Close Contacts in Nanjing, China. Science China Life Sciences. 2020;63:706–11. Available from: `https://doi.org/10.1007/s11427-020-1661-4`.

7. Bajema KL, Oster AM, McGovern OL, et al. Persons Evaluated for 2019 Novel Coronavirus — United States, January 2020. MMWR Morbidity and Mortality Weekly Report. 2020;69(6):166–70. Available from: https://www.cdc.gov/mmwr/volumes/69/wr/mm6906e1.htm.

8. Jiehao C, Jin X, Daojiong L, et al. A Case Series of Children With 2019 Novel Coronavirus Infection: Clinical and Epidemiological Features. Clinical Infectious Diseases. 2020;71(6):1547–51. Available from: https://doi.org/10.1093/cid/ciaa198.

9. Faes C, Abrams S, Van Beckhoven D, et al. Time Between Symptom Onset, Hospitalisation and Recovery or Death: Statistical Analysis of Belgian COVID-19 Patients. International Journal of Environmental Research and Public Health. 2020;17(20):7560. Available from: https://dx.doi.org/10.3390/ijerph17207560.

10. Chu DK, Akl EA, Duda S, et al. Physical Distancing, Face Masks, and Eye Protection to Prevent Person-to-Person Transmission of SARS-CoV-2 and COVID-19: A Systematic Review and Meta-Analysis. The Lancet. 2020;395:1973–87. Available from: https://doi.org/10.1016/S0140-6736(20)31142-9.

11. Brainard J, Jones NR, Lake IR, Hooper L, Hunter PR. Community Use of Face Masks and Similar Barriers to Prevent Respiratory Illness such as COVID-19: A Rapid Scoping Review Eurosurveillance. 2020;25(49):pii=2000725. Available from: https://doi.org/10.2807/1560-7917.ES.2020.25.49.2000725.

12. Altman D, Kaiser Family Foundation. Most Americans are Practicing Social Distancing. Axios. 2020. Accessed: 2020-06-21. Available from: https://www.axios.com/coronavirus-social-distancing-lockdown-polling-7c27d86f-bb4b-4cbf-aedf-cfdd26799fd1.html.

13. Tian T, Huo X. Secondary Attack Rates of COVID-19 in Diverse Contact Settings, a Meta-Analysis. The Journal of Infection in Developing Countries. 2020;14(12):1361–7. Available from: https://doi.org/10.3855/jidc.13256.

14. Madewell ZJ, Yang Y, Longini IM, Halloran ME, Dean NE. Household Transmission of SARS-CoV-2: A Systematic Review and Meta-Analysis. JAMA Network Open. 2020;3(12):e2031756. Available from: 10.1001/jamanetworkopen.2020.31756.

15. Lauer E, Houtenville A. 2017 Annual Disability Statistics Supplement. Institute on Disability, University of New Hampshire. 2018. Available from: https://disabilitycompendium.org/sites/default/files/user-uploads/2017_AnnualReport_FINAL.pdf.

16. U S Bureau of Labor Statistics. Employment Projections. 2019. Accessed: 2020-10-31. Available from: https://www.bls.gov/emp/tables/emp-by-detailed-occupation.htm.

17. McNicholas C, Poydock M. Who are Essential Workers? Economic Policy Institute. 2020. Accessed: 2020-10-31. Available from: https://www.epi.org/blog/who-are-essential-workers-a-comprehensive-look-at-their-wages-demographics-and-unionization-rates/.

18. United States Census Bureau. National Population Totals and Components of Change: 2010–2019. 2020. Accessed: 2020-10-31. Available from: https://www.census.gov/data/tables/time-series/demo/popest/2010s-national-total.html.

19. Statistics Canada. Census Profile, 2016 Census. 2019. Accessed: 2020-06-21. Available from: https://www12.statcan.gc.ca/census-recensement/2016/dp-pd/prof/details/page.cfm?Lang=E.

20. Rothwell J. Americans' Social Contacts During the COVID-19 Pandemic. Gallup Blog. 2020. Accessed: 2020-10-31. https://news.gallup.com/opinion/gallup/308444/americans-social-contacts-during-covid-pandemic.aspx.

21. Mossong J, Hens N, Jit M, et al. Social Contacts and Mixing Patterns Relevant to the Spread of Infectious Diseases. PLoS Medicine. 2008;5(3):e74. Available from: https://doi.org/10.1371/journal.pmed.0050074.

22. Rothwell J. A Survey of Essential Workers Shows a Political Divide. The New York Times. 2020. Available from: https://www.nytimes.com/2020/04/27/upshot/red-blue-workplace-differences-coronavirus.html.

23. Ottawa Public Health. Daily COVID-19 Dashboard. Ottawa Public Health; 2020. Accessed: 2020-08-24. Available from: https://www.ottawapublichealth.ca/en/reports-research-and-statistics/daily-covid19-dashboard.aspx.

24. Fosdick BK, Larremore DB, Nishimura J, Ugander J. Configuring Random Graph Models with Fixed Degree Sequences. SIAM Review. 2018;60(2):315–55. Available from: https://doi.org/10.1137/16M1087175.
