# Supplementary material for: Networks of necessity: Simulating COVID-19 mitigation strategies for disabled people and their caregivers
Source: PLoS Comput Biol. 2022 May 18;18(5):e1010042. doi: 10.1371/journal.pcbi.1010042 (PMC9232173; doi:10.1371/journal.pcbi.1010042)
Supplement: Attachment — Submitted filename: ResponseToReviewersFinal.pdf [file pcbi.1010042.s002.pdf]

Dear Jacopo and Nina,

Thank you for handling our manuscript. We are pleased by the overall positive reactions and critiques of the referees, and we appreciate their time (and yours) in handling our paper. We hereby submit our revised manuscript. We have addressed the referee comments thoroughly and provide point-by-point comments below.

Sincerely,

Mike Lindstrom
(on behalf of all authors)

Dear Reviewers,

We thank the reviewers for taking the time to read our manuscript and providing us with constructive criticism and insightful questions about our work.

Response to Reviewer 1:

Thank you for your detailed reading of our manuscript and for your comments on our parameters and the variation in epidemic outcomes, and for helpful feedback in improving the presentation.

I think that the epidemiological times involved in the transitions from A to I compartments and from I to R are too long. Regarding the first one, the authors assume that once they individuals are infected, they are also infectious and spend a given time before developing symptoms, which is adapted from the median of the distribution of incubation periods reported in [64]. Actually, the infectious period before being symptomatic is always shorter than the incubation period since the viral load is not high enough at the early stages to allow them to transmit the virus. Furthermore, although from a clinical point of view, it takes 14 days to recover from COVID-19, the infectiousness of symptomatic individuals sharply decreases around 5 days after showing symptoms, being the latter the relevant time for the model. To compensate

the long infectious periods, it seems the calibrated transmissibility (0.01-0.02) is lower than the one used/calibrated in other studies like [Pullano et al. Nature 590 (2020)] or ref. [38] (0.07-0.08). I would like the authors to comment the differences of the parameters with the ones reported in the literature and also the possible influence of the longer epidemiological times on the epidemic trajectories generated by the model. I guess that including a model with shorter infectious periods would allow the system to respond more sharply to containment policies, thus better capturing the shape of the data.

Response: We have added an Exposed compartment to our model, and we have also re-estimated many of the transition rates based on newly published data. In particular, individuals spend a mean of 1 day being exposed before moving into the asymptomatic compartment, they spend a mean of 10 days asymptomatic before becoming ill or removed (compared to 7 days before), and they spend a mean of 12 days ill before moving to the hospital or being removed (compared to 20 days before). The justification appears in part A of our Supporting Information.

We appreciate your comment on the infectiousness dropping sharply after 5 days and recognize that this could have a significant impact on the results. However, we were unable to find your claim substantiated in the literature. Indeed, papers that presented such studies often had durations of 12 days or longer; see the survey article of Byrne et al. that we cite.

We also changed our metric of transmissibility from a rate to a probability (based on another reviewer's comments) but, interestingly, the probability that we estimate is still about 0.01. To arrive at this value, we used data on secondary attack rates and the compartment transition rates that we inferred from the literature (instead of fitting for β with Ottawa data, as we did in the original version of the manuscript).

We speculate that the smallness of β in comparison to other models is a result of our network structure incorporating "heavy tails" in degree distribution, with certain individuals having many more contacts than others. This allows the disease to spread more effectively. Indeed, as we note in our response to your next query, the heterogeneous degree distribution gives rise to a large variance in epidemic trajectories.

We also note that the value of $\beta \approx 0.07$ in the cited *Nature* paper is inconsistent with other studies on secondary attack rates. For example, if

$\beta = 0.07$ and if we assume that a person is infectious for only 5 days (likely a lower bound), then the probability that they infect a given family member is $1 - (1 - 0.07)^5 \approx 0.3$, which is larger than the 20% in the study we used focusing directly upon secondary attack rates within households. In short, our value of β is appropriate for our model and for the supporting literature.

In paragraph 3 of the Results section, we now discuss the discrepancy between our employed β value and other β values.

The variance of the results presented by the authors when performing the stochastic simulations is remarkable. Does this variance comes from the power-law nature of the distribution of weak contacts across the population?

Response: This is a good question. As a result of your asking this, we have investigated the matter. The large variance does indeed appear to arise from the heavy-tailed nature of this distribution. When we used a 'fixed' distribution, in which each person of a specific subpopulation (caregiver, disabled, essential, or general) has a fixed number of weak contacts that matches the mean number of contacts in the truncated power law, the epidemic hardly spreads and the variance is significantly smaller. The potential for "superspreader" events was one of our our motivations for choosing to use a heavy-tailed distribution in the first place; the consequence is that there is a larger variation in the outcomes.

We added this remark in Paragraph 2 of the results and section C of the Supporting Information provides further details.

The assessment of the vaccination strategies involving different groups is really interesting and clearly reveal the relevance of central agents/node (caregivers) in the network. However, they mostly rely on assuming that vaccination completely removes agents from the system which allows for break down the transmission chains connected by caregivers. While there is a global consensus on the efficacy of the vaccine in preventing severe/mild infections and despite some promising preliminary results, it is not totally clear whether COVID-19 vaccines significantly reduce the infectiousness of vaccinated individuals. I think that the authors should include a discussion on this topic as a possible limitation of the results of the manuscript.

Response: The prescience of this comment is remarkable, especially given the emergence of the delta variant and that we submitted our manuscript in January. It seems clear that the vaccine still provides some degree of protection from infection and near-total protection from hospitalization and death, although this may change with future variants. In December 2020, when we were finishing up the original version of the manuscript, we also did not appreciate the difficulty of achieving a 100% vaccination rate among health-care professionals (such as caregivers for disabled people). Because neither vaccination rates nor vaccination efficacy are perfect, we have chosen to focus on the scenario in which an equivalent of half of all caregivers are vaccinated with total effectiveness. We agree that we cannot claim to be modeling any particular real-life scenario with this, especially given how rapidly the virus and vaccination rates are evolving, and we have noted this in our revised discussion (including in our discussion of our work's limitations).

As minor details:

- I would include in Fig. 4b a new panel comparing the new daily positive test according to both data and the model. In addition, for the sake of readability, I would use the date in the x-axis rather than the total number of days.

Reponse: Thank you for this suggestion. We now have both new daily cases and cumulative cases in Fig. 4. We have also added the dates.

- It would be very illustrative to indicate in Fig. 2 who correspond with the weak and the strong caregiver respectively.

We have added a label to indicate the weak caregivers.

- I would rewrite of the paragraph indicating the higher susceptibility of Black and Hispanic people to COVID-19 to stress that it is related to the social circumstances usually affecting them rather than to their ethnicity per se; otherwise it can be misinterpreted.

Response: Thank you for pointing this out. We agree with you and have added a clarification to this sentence that systemic disadvantages in accessing

healthcare leads to the increased COVID-19 morbidity and mortality among Black and Hispanic populations (see paragraph 4 of the introduction). We also added a clarification to a similar point in our Discussion section.

- The authors should revise the references to check if the preprints have been already published. For example, ref. 38 should be updated, since the manuscript is already published in [Physical Review X 10 (4), 041055 (2020)].

We have updated all references for which we found published or otherwise updated versions.

Response to Reviewer 2:

Thank you for your detailed reading of our manuscript and for your questions about the choice of model and parameters.

The work proposes a model for the spreading of COVID-19 among disabled people and their caregivers. The authors implement a detailed agent based model with four different types of agents (general population, essential workers, caregivers and disabled people) also including the effect of non-pharmaceutical interventions like the use of masks and social distancing. The main findings are that the caregivers-disabled community is extremely vulnerable to COVID-19 and vaccination strategies aimed directly at caregivers would be highly effective in protecting both disabled people and the general population (even more than vaccinating disabled people directly).

Response: This is an accurate summary of our work.

The topic could be of some interest and the work focuses on a category (home caregivers) overlooked in the thousands of works published this period on COVID-19 epidemiology. Having said that, however, there are some points that make me doubt about the relevance and possible impact of the results.

Response: We agree that the topic is of interest and that it has been overlooked in the literature in the thousands of papers on COVID-19. That is why we felt it was important to study it ourselves.

Here there are my major and minor remarks:

- From the plethora of studies published in the last year about COVID-19 diffusion it is clear that the extremely variable latency period and prodromic infections play a crucial role in the spreading (see for example Ferretti et al. "The timing of COVID-19 transmission" medrxiv) delaying identification and blueucing the effectiveness of interventions. Thus, is quite strange that a model so complicated in the representation of interaction networks employs a relatively unrealistic epidemic dynamics. Although I do not believe that the essence of the results would change, my suggestion is to move to a more realistic epidemic model (e.g. the one in Di Domenico et al. BMC Med 18, 240 2020) that includes, at least, exposed and prodromic infections.

Response: When modeling real-world scenarios, some assumptions and simplifications are warranted to make the models more tractable and interpretable. Making all elements of the model extremely detailed (and complicated) makes it very difficult to separate what results come from what assumptions. Because our work focuses on the types of interactions and their relative risks, we chose to simplify the compartments to the extent possible and thus used a reasonably simple, but qualitatively accurate, set of compartments while being more detailed in the types of people in society and their interaction risks. This choice reflects the focus of our paper.

However, based on the referee's suggestion, we added realism to our set of compartments by adding an Exposed compartment. In the Exposed compartment, individuals cannot yet spread the disease. They can then move from Exposed to the Asymptomatic compartment, which effectively includes prodromal infections, asymptomatic, and mildly symptomatic situations (which are all situations in which a person does not know that they are sick). A remark has been made clarifying this in our manuscript in paragraph 3 of section 2.1. We thus now have compartments with exposed and prodromal individuals, as you suggested. We have also redone our parameter estimates based on newer studies. For full details of the revised parameter estimates,

see Section A of our Supporting Information.

As you correctly anticipated, the essence of our results do not change, but we are happy to have a more realistic model.

- Along with the epidemic dynamics, I am also puzzled by some of the results presented in the manuscript:

- First of all, the large variability in the model runs when comparing with the real data (Fig.4). Simulations show extremely large confidence intervals even during the calibration phase (till day 97). Intervals should be quite small during calibration and become larger after that. This is quite strange especially considering that the authors are fitting four parameters. The causes could be, among many others: a model not able to fit the data (see my comment above), a poor choice of the parameters for the fitting or the fact that parameters are changing over time.

Response: We have investigated the large variability in addressing comments by Referee 1. Much of the variability seems to be explained through the heavy-tailed distribution of weak contacts, which is appropriate to allow some individuals to have many more more contacts than others. Additionally, we have redone our parameter fitting so that it now has only 2 degrees of freedom plus a constraint (to set 3 parameters). In the original manuscript, we had 3 degrees of freedom plus a constraint (to set 4 parameters). We now estimate β from secondary attack-rate data in the literature, rather than through Ottawa-specific data.

The only parameters that we now estimate by fitting are the probability τ that a symptomatic person is tested and the maximum number C^* of weak contacts that a individual can have. The variance is much smaller with these new fits. At the end of calibration, the range of uncertainty is roughly from 2000–3000. (In the original manuscript, this range was 1000–3500.)

While obtaining a better fit is possible, it would likely come at the expense of overfitting, which we wish to avoid. With respect to the choice of parameters, every city could have specific parameters that are tailored to that city and allowed to vary over time. However, this would lead to overfitting. We have tried to find and estimate as many parameters as possible directly from the

literature specifically so that we can fit for as few parameters as possible in our study. Given our limited degrees of freedom, we believe our results are strong. This remark on how accurate our epidemic curves are despite limited degrees of freedom has been made in the first paragraph of our Results section.

- Along the same line, I have some doubts about the choice of fitting the parameters over a period that covers both the early phases of the outbreak (without restrictions) and government interventions that drastically change evolution of the disease (i.e. the inflection point in the data around day 80). Some of the parameters could change between the two phases making the fitting more complicated. Thus, my suggestion is to try to fit only the first part of the data or, at least, limit the fit to day 60 or 65.

Response: Thank you for this suggestion. As a result of your question, we tried fitting over shorter windows of time but found that fitting over these shorter time windows yields poorer fits. We have only reduced the fitting window to 90 days from 97. The likely reason for the poor fitting on shorter time windows is that the parameter C^* is smaller when fit over shorter intervals (because the epidemic has spread less at that point). The longer time window allows C^*, which may be a key driver in the epidemic, to be fit to a larger value.

We have added this commentry to the third paragraph of part A.2 of the Supporting Information as well.

- Another point is about the number of first and second neighbors (and strength) in Fig. 5. I think this is crucial to assess the relevance of the work as the very large average degree of caregivers can explain all the results of the work: it is well known in the computational epidemiology literature that hubs in scale networks are more vulnerable than other nodes and several immunization strategies are based on identifying and vaccinate hubs first. What puzzles me is the huge difference in degree (more than double) between caregivers and the general population or essential workers and, even after reading three times section A of the SI, it is not clear to me if the difference is really present in the data (i.e. in reality caregivers have way more contacts than the rest of the population) or it is an artifact of the assumptions taken to build the network.

My opinion is that the authors should try to clarify this point by testing different ways of building the networks or analyzing some type of demographic data. I understand that such analyses could be difficult and data hard (or even impossible) to find but, as I said, I find really strange that caregivers in real life have on average more than the double of contacts that essential workers or any other person. Moreover, something similar also happens for disabled people.

Response: Thank you for pointing this out; we made an error when preparing the figure. Instead of including contacts on an example day, we included all of the possible contacts for all of the possible network nodes. Because caregivers contact only a small portion of their designated pool of disabled people per day, this artificially inflated the number of contacts that the caregivers have. We have fixed the issue by plotting the number of contacts per day instead of over all time. We appreciate your calling attention to this error.

- The manuscript is well written and the presentation is, in general, clear. However, I have to admit it took me three readings of sec. 2 and the SI to understand the (quite complicated) model and still I am not sure I got all the details. I acknowledge that the authors tried to give enough details in the main text (e.g. in section 2.2) but my suggestion is to try to clarify more the network creation process that, right now, is quite confusing.

Response: Thank you for this feedback. We understand there are a lot of details in the network construction and that it can lead to confusion. We have modified Sec 2.2 (notably paragraph 2) to improve our exposition of the network structure and have added a few paragraphs in the SI to give a high-level view of the network creation (see paragraphs 2-5 of B.1).

Response to Reviewer 3:

Thank you for your feedback on our work and for asking questions about our model and assumptions. This has allowed us to improve our presentation and model justifications.

Simulating the spreading of COVID-19 in different scenarios has become a major necessity to understand the dependencies between the different intervening factors, and to be able to give informed advice to the health authorities. In this paper, the authors focus on the role of disabled people and their caregivers as spreaders and targets of COVID-19. This is important since disabled people cannot live without the support of their caregivers, and usually they are considered as highly vulnerable to the disease. Unfortunately, caregivers can also be the vectors of the disease, thus compromising their attendees. This work analyzes this kind of interrelation at the level of a city, with people that can be: disabled, caregivers, essential worker, and general population. Data from the city of Ottawa (Canada) has been used to establish the setup and calibrate the model: structure of the population, evolution of the pandemic, and non-pharmaceutical interventions (NPI). The result is a series of simulations which cover a large set of scenarios, which evaluate the impact of the different interventions and possible people behaviors on the selected collectives. Finally, the paper tries to find the best vaccination strategy.

The paper is very well-written and organized, the figures and tables are clear to convey the messages and results, and the methodology is in accordance with the standards for this kind of scientific works. However, since the simulations are trying to reproduce a real situation, and all the results depend on a large number of parameters and assumptions, we must be sure that all the important details cannot be disputed.

The epidemic model, though minimalist, is enough for the object of study and, to the best of my knowledge, all the selection, decision, and calculation of the parameters of the model are acceptable, in accordance with the current literature. Of course, there exists an important uncertainty regarding some of the parameters, but in general the selections could be considered as correct. Anyway, I have serious doubts on one of the parameters, which potentially could lead to important differences in the results, and on the quality of the calibration.

Response: This is an accurate summary of our work. Thank your for appreciating our efforts to communicate clearly. We address your concerns below.

Let me start with the main issues:

1) I do not understand why you use weights in Eq. (1) instead of rates or

probabilities. With weights, beta loses its traditional meaning of infection rate per contact, becoming a combination between the infection rate and a normalization constant. However, if beta were an infection rate, you could recover its value from the literature instead of relying in fitting it.

Response: Based on this suggestion, we have changed our definition of β. It is now the probability of transmission from a contagious person to a susceptible person during a strong interaction and without a mask. Additionally, we now determine β based on secondary attack-rate studies, independently from the Ottawa data. See Section A.1 of the Supporting Information.

Our β value is smaller than other values in the literature. We anticipate that this comes from (a) the heavy-tailed distribution of weak contacts, which allows superspreader events; and (b) the duration of infectiousness that we inferred from the literature. Indeed, as we remark in our response to Reviewer 1, using values of β directly from other papers is inconsistent with data on secondary attack rates.

Many existing studies use models to infer parameters, but those parameters may not be appropriate as a starting point for other models. We sought to infer parameters directly from clinical and empirical data, rather than base our parameters on other people's models.

2) My main concern is with the weight for the caregivers. It has been set to 1.7, which means 70% higher than for household contacts. You just point to reference [62] but, looking at that reference, it is not easy to infer what have you done to assign this value. Moreover, I simply do not agree in assigning a larger weight to caregivers than to household contacts. I believe there is no possible reason for that. Given that COVID-19 is airborne (the evidence for this fact is overwhelming), and disabled people are going to have the contacts with household and caregivers' counterparts usually in scarcely ventilated indoor places, the risk of getting infected is going to be equivalent in both cases. Household contacts should be the upper bound for infectious risk, since people at home do not wear masks, share the air for long times, and no social distance is maintained. Maybe there is a problem with the interpretation of the data in [62]. A larger secondary attack rate for caregivers should come from a larger number of contacts, not a higher risk of contagion, which is what should appear in Eq. (1). The only remaining factor to consider, the

duration of the contact, should also be larger for household contacts than for caregivers.

Response: Here we need to push back, as the referee may have some misconceptions about interactions between caregivers and disabled people. Caregiving work is extremely intimate and requires extended, close physical contact and potential exposure to bodily fluids. Such a level of intimacy is not typical between housemates. Therefore, we find it entirely appropriate to give larger weights to caregiver–disabled contacts than to household (i.e., strong) contacts and certainly than weak contacts, such as those between the general population and essential workers. In the absence of designated data about caregiver–disabled transmission, we have chosen to use spouses as a proxy for the level of intimacy of caregiver–disabled contacts.

Because it is likely that others who read our work could have the same initial misconception, we have added paragraph 2 of the "Baseline Transmission Probability β and Caregiving (w_{c}) and Weak (w_{w}) Edge Weights" in section A.1.2 in which we estimate β, w_w and w_c to explain this choice.

We also wish to clarify an important point that we may not have expressed clearly in our original manuscript: with masks on, when caregivers and disabled people interact, the transmission risk is actually lower than between family members because masks are not typically worn within the house. The relative risk of $w_w = 1.7$ (and is now $w_w = 2.27$ based on our new fits) describes the relative risk of transmission between caregivers and disabled people to transmission between housemates under the assumption that nobody is wearing a mask.

3) In view of my previous concern, I would suggest to also check the value assigned to weak contacts.

Response: We have refit the weak and strong contact weights based on the secondary attack-rate data. The existing literature supports the fact that weak contacts have a lower risk than household contacts, which in turn tend to have a lower risk than spousal contacts. We show the details in Section A.1 of our Supporting Information. Paragraph 3 of the "Baseline Transmission Probability β and Caregiving (w_{c}) and Weak (w_{w}) Edge Weights" section,

in particular, explains how the weights are assigned based on data from the literature.

4) I would say that most of the obtained results heavily depend on this assignment of such a large weight for caregivers, thus the conclusions could require big adjustments.

You raise a good question, so we have investigated this matter. We found that our results do depend on the caregiver–disabled weight w_c, but not to a large extent. With $w_c = 1$, essential workers seem to spread the disease more than other subpopulations. However, as we have discussed above, it is very reasonable that $w_c > 1$ because of the intimacy of interactions. For example, suppose that $w_c = 1.5$ (instead of the $w_c = 2.27$, as in our newly fit parameters), caregivers spread the disease to disabled people more than the other subpopulations. Therefore, the essence of our message does not change even with a smaller weight.

We have added these results to the Supporting Information (in Section C) and to our Results section in the paragraph that starts with "As we explain in the Supporting Information (in section A), because of the intimacy ..."

5) The deviation between data and simulations (Fig. 4) is quite large. If instead of plotting the accumulated cases (not recommended) you plot the new daily cases, the deviations would probably be more evident. In general, for a good calibration of the model, you need good data. It is well-known that, in the initial phase of the pandemic of COVID-19, the capacity of testing was very low in most countries, and only people with symptoms were detected; remember that for some time it was even argued that asymptomatic COVID-19 was not possible, and if so, some believed they could not be contagious. This means that the real number of cases could have been much larger than the detected through the scarce number of tests, thus compromising the calibration of the model. I do not know if this happened in Ottawa, but at least it must be taken into consideration. I would have expected a large underestimation of new cases at the beginning, but good agreement at the end. This is not what we observe in Fig. 4.

Response: The referee raises a valid point. In the original manuscript, we

used the parameter τ to account for the lack of testing. We made a typo in stating "We also assume that only small fraction τ of asymptotic individuals are tested, but that all hospitalized individuals receive positive test results for COVID-19." We should have written "We also assume that only small fraction τ of **symptomatic** individuals are tested, but that all hospitalized individuals receive positive test results for COVID-19." Our assumptions were that a symptomatic person who is not hospitalized will get a positive test with probability τ and that a person who is hospitalized for COVID-19 will automatically get a positive test if not already diagnosed. Indeed, as can be noted by the large number of initial infections (about 140 in the original manuscript and about 340 in our revised manuscript) with only a single reported case on day 1, there is indeed substantial underreporting at the beginning of the pandemic.

Inspired by this comment on new daily cases, when refitting our parameters, we fit against new daily positive cases rather than cumulative positive cases. Thank you for this idea.

6) A better calibration approach could have been the use of the new daily hospitalizations or new daily deaths instead of new cases, since those data are much more reliable. Unfortunately, the model merges fatalities with recoveries, thus making impossible to use deaths for its calibration and/or validation, and probably daily hospitalizations would require to separate ICUs from non-ICUs, depending on the data availability.

Response: We disagree that hospitalizations or deaths would be better for calibration. Hospitalization and death both require an individual to pass through multiple stages of the disease, thereby possibly increasing the uncertainty in using these values. This is exacerbated by the fact that hospitalizations and deaths are highly age-dependent, which would contribute additional variables to our model. Moreover, as we mentioned above, we were already taking into account the underreporting with the parameter τ.

We also note that in our new manuscript, we fit β independently from the Ottawa data, so we now fit our case data with only 2 degrees of freedom (i.e., one less than before), improving the reliability of our parameter estimates.

The summary of the previous issues could be that any conclusion on COVID-

19 requires a good calibration and fitting of the parameters of the model, and I have serious doubts on them.

Response: We believe that our responses above have addressed the concerns that have been raised.

Other minor issues:

1) The introduction is now a bit old: currently, there are several vaccines available for COVID-19. An update of the whole text to the current situation would be a good idea, even if the paper is limited to the analysis of the first wave.

Response: We agree with the reviewer and have removed material in the Introduction that has become outdated since the end of December 2020, when we submitted our manuscript for review. We have also added a paragraph to discuss the widespread availability of vaccines in many countries. We nore that our findings on how to effectively utilize limited vaccine doses are still relevant to countries in which vaccines are being rolled out more slowly and in the event of possible future pandemics. We believe that other points in the Introduction, such as the increased vulnerability of disabled and caregiver populations and the need to assess both non-pharmaceutical interventions and vaccination strategies, remain relevant. We appreciate the opportunity to update our work.

2) It would be helpful to establish the correspondence between dates (10 February, 24 March, etc.) with the number of days that appear in the plots (1, 44, etc.).

Response: This is a good suggestion. We have done so.

3) Section 2.2: "essential workers either do or do include mask-wearing" → "essential workers either do or do NOT include mask-wearing"

Response: Thank you for noticing this. In our revised version of the manuscript, this sentence has been reworked entirely.

4) Please specify the NPIs used in Fig. 4. Is it D+C+E after day 44?

We now specify the NPIs in the figure caption.

5) When describing the structure of the network before and after the lockdown, it is not mentioned the specific days selected. I guess the structure is statistically equivalent for all the days in the same interval.

Response: This is correct. The specific day does not matter. We have noted the specific days that we used for the figure (day 43 before distancing and day 45 during distancing) in the figure legend.

6) All the main issues described above arouse after looking at Fig. 5, in which the strength of caregivers and disabled are too different with respect to general and essential, impossible to understand and believe.

Response: See our response for major concern item 2.

7) The criteria that disabled population has the same mean value for weak contacts as the general population seems an overestimation: it seems more realistic that disabled people has less contacts.

Response: We searched for data on this and were unable to find detailed quantifications for of number of social contacts that are enjoyed by disabled versus non-disabled adults. In looking at these data, we see that the longitudinal data does support a general reduction in the number of social events that are attended by elderly people who acquire a physical disabililty versus those who do not. However, these interactions are complex and depend on the nature of the social relationship (e.g., kin versus non-kin), the gender of the person who becomes disabled (e.g., men tend to lose social contacts, whereas

women do not), and the nature of the disability. (See Anderson, Falci, and Warner (2015), Physical Disability and Changes in Older Adults' Social Network Ties: A Longitudinal Examination of Ego-Alter Dyads.) However, the cited study focused on the elderly and thus does not capture the full set of disabled people. We also note that disabled people who employ caregivers for bathing, getting dressed, and so on are not necessarily homebound. Two of our coauthors employ caregivers while holding full-time jobs. They both have extensive numbers of contacts and active social lives.

8) In Fig. 12A, a dashed line on top of a solid line (with different widths) would allow to see both curves.

Response: Thank you for the suggestion. We have done so.

9) Please check the references, some of the preprints could have already been published.

Response: We have updated our references to their published forms when relevant.
