# Supplementary material for: Networks of necessity: Simulating COVID-19 mitigation strategies for disabled people and their caregivers
Source: PLoS Comput Biol. 2022 May 18;18(5):e1010042. doi: 10.1371/journal.pcbi.1010042 (PMC9232173; doi:10.1371/journal.pcbi.1010042)
Supplement: Attachment — Submitted filename: ResponseToReviewers.pdf [file pcbi.1010042.s003.pdf]

Dear Jacopo and Nina,

Thank you for handling our manuscript. We have taken the occasion to make final adjustments and improvements to our manuscript based on the reviewer feedback. We hereby submit our revised manuscript.

Sincerely,

Mike Lindstrom
(on behalf of all authors)

Dear Reviewers,

We thank the reviewers for an additional reading of our revised manuscript and for the additional comments.

Response to Reviewer 1:

The authors have addressed correctly my concerns and I therefore recommend this article for publication in PLOS Computational Biology.

Response: Thank you for the recommendation.

Response to Reviewer 2:

Let me start by saying that I really appreciated the efforts made by the authors in trying to address my and the other reviewers comments. The work is now clearer and surely more solid. The manuscript went through a profound revision and now the many assumptions behind the model and the network are clearly stated. I also appreciate the choice of the authors to move to a more realistic epidemic dynamics that would help grasping the timescale of COVID-19 spreading.

Thank you for noting the improvements in our manuscript. We are pleased to hear the model assumptions are now more clear.

Said that however, I think that some of the main issues raised by me and the other reviewers in the previous round have not been fully addressed (and, more importantly, I am not sure it would be possible to address them).

Summarizing, most of the results presented rely heavily on three main points:

- The epidemiological model that, in order to be able to make reliable predictions, should reproduce the dynamics of the disease.

- The fraction of individuals and the number of their contacts for the four categories considered.

- The weights assigned to each type of contact.

The problem is that all these points depend on assumptions/estimations that are hard to test or too sensible to changes/errors in the data.

We address your points above in responding to your more detailed comments below.

Being more specific:

- The fitting of the model is still too poor to assure that it is able to reproduce CODIV-19 evolution. I understand the difficulties and I think the authors did their best to solve the issue however, given the huge differences between the curves in fig.4, I do not think that the model is capable of reproducing the dynamics of the disease (I am referring not only to the still quite large variability, but also to the differences in the final number of infected, etc.).

The aim of our model was to be qualitatively accurate. The real world data is itself quite noisy, with additional errors coming from things like delays in reporting. Additionally, the virus has mutated several times and thus many parameters, such as infectivity, evolve with time. Therefore, the only real way to accurately fit the case data would be through overfitting and adding many assumptions and corresponding parameters that are difficult to estimate. We also argue that, in light of the uncertainty in "ground truth" data, having a qualitatively accurate model is significantly more useful. Our model produces the typical cumulative infections curve of a traditional SIR model, with similar peak infections. Additionally, as we consider through the sensitivity analysis, even in varying some of the parameters which have the highest uncertainty, our main results are preserved. Overall, we do not believe that a more accurate fit would be beneficial. Furthermore, we believe

that it could result in the all-too-common data-science error of overfitting.

Along with many others, the cause could be in the reliability of the data. In this sense, I strongly support the suggestion of Reviewer 3 of using hospitalization and deaths data instead of case counts (a solution widely adopted in the literature) as, even if they could bring some biases (age differences and so on), they are way more solid than infection counts.

On this point, we respectfully disagree. Even if this is commonly done in the literature, this does not mean that it is a good idea — at least, not in our view. Here's a simple analogy for why counting hospitalizations or deaths would be inadequate. Consider a mix of marbles of different colors — equally mixed between red, yellow, and blue. Suppose that we study these mables after placing them through a filter. Absent any other knowledge, we could estimate how the filter works by studying the distribution and number of marbles that come out. But if someone else were actually stealing all of the red marbles right after they got out of the filter, we might incorrectly estimate how the filter is working. If one were to replace the observed marbles with hospitalized cases, the filter with actual COVID-19 infections, and the stealing with groups of people less likely to be hospitalized, then the problem is clear (there could be many more active cases than hospitalization data would suggest) and our study is concerned primarily with how many people have COVID-19.

It is plausible that epidemic curves would be considerably different when initial infections are predominantly in younger populations. In such a situation, it seems more appropriate to base active infections on positive test cases while accounting for limited testing. Additionally, because the hospitalization data are sparser than other available data, using such data could result in fitting artifacts rather than genuine trends.

Another problem could be in the parameters they are trying to fit and not in their number. I am not sure that the probability of being tested ill and the maximum number of contacts are the best choices. A common practice in the literature is to employ a metapopulation model (so a well-mixed population at the lowest scale) and fit only one parameter, representing the transmission probability. A possible way of adapting this line of reasoning to the contact network proposed by the authors could be to fit the baseline infection probability and one of the weights used to model the contacts but, this is just a

suggestion.

We used secondary attack rate data and selected β and the weights to match that data. With this method, we fit one parameter to estimate the transmission risk; this fitting is independent of our network simulations.

With respect to the probability of being tested if ill and the maximum number of weak contacts, those were parameters in our model and we needed to estimate them. It would be very hard to justify not including these values in our model because it is well-understood that testing was a limitation at the start of the pandemic and it is unrealistic for a single person to have close to 1 million contacts in a day. We found nothing in the literature to infer these parameters, so we obtain them by fitting.

We recognize that a metapopulation model is another approach. It is not the study that we did. We would welcome other authors to build their own metapopulation models or to employ complementary approaches.

- The network building algorithm depends heavily on the estimation of the number of strong and weak contacts that, although based on Canadian census data, could be unreliable. For example, starting from data in refs 19 and 20 of the SI, the authors estimate that each disabled individual would see, on average, 2 caregivers. The authors then calculate O_c as the fraction of disabled people divided by the fraction of caregivers and multiply by 2, getting $O_c = 6.95$. Even if this value is way more reasonable than the one in the previous version of the work (due to a plotting error), it is still 1.7 contacts per day larger than the regular population and I still cannot find a reason for that. More importantly, given the small fractions of caregivers and disabled people, small errors in their number (the number of caregivers could be easily underestimated due to illegal work, etc.) could lead to quite large changes in O_c. This limitation has been correctly highlighted by the authors in the conclusions, however, I do not think that stating it is enough. A sensitivity analysis for most of these estimates should be done to check the robustness of the results.

Based on this comment, as an additional component of sensitivity analysis, we varied the fraction of the population who are caregivers to consider the case when caregivers have the same number of occupational contacts as the general and disabled subpopulations. We show the results in Fig. 9. The numbers do change a little, but there is no change in the order of the fractions

of each subpopulation that become infected.

- A similar line of reasoning holds for the weights of the different interactions (as pointed out by Reviewer 3). In their response to the comments raised by the Reviewer, the authors provide a compelling argument for assigning larger weights to caregivers interactions and I appreciate the sensitivity study conducted in this sense. However, there is little support to this assumption in the data or in the literature. Again, I trust the narrative proposed by the authors, but I think that more evidence is needed to justify it and small differences in these estimates could lead to larger errors in the results.

Finally, all these sources of uncertainty combined make me doubt the solidity of the results.

We acknowledge there are presently no data to pin down the appropriate weights. However, we have shown through the sensitivity analysis that our main results (that caregivers are highly central in the spread of COVID-19, especially to disabled people) do not change. See Fig. S3.

We are opting to publish the peer-review history for this article. In describing the limitations of our model, we encourage readers to read the referee commentary along with our rebuttals.

Minor remarks:

- In eq.1 σ *should be* σ_i

Thanks!

- On page 12 when discussing Fig. 5 the text states: "We find that caregivers have the most first-degree and second-degree contacts (see Fig. 5A,B)" while from the figure it seems that essential workers have the larger number of contacts. Probability this is an error from the previous version of the manuscript.

Thanks for noting this. We have corrected it.

Concluding, although the authors made a huge work in spelling out all the assumptions and limitations of their model, my overall impression is that it relies too heavily on them to support solid conclusions.

We appreciate your commentary on our work and your skepticism of some of our results due to your continued concerns. While it seems that we will

have to “agree to disagree,” we believe that scientific dialogue is important. Therefore, we have elected to publish the peer-review history so that your criticisms will be available for readers. We reference the report on page 21.

Response to Reviewer 3:

The authors have made an extraordinary work dealing with my comments and those of the other reviewers, carefully addressing even the most secondary issues, and with a great scientific rigor. For sure, I can grant publication as is, and I apologize for the delay I could have added to the review process.

Minor comment: It would be useful that the Table and Figures in the Supporting Information were indexed as S1, S2, etc., to avoid confusion with the Table and Figures in the main text.

Response: Thank you for your positive endorsement. We have also followed your suggestion in labelling the figures and tables of the Supporting Information.
